# How to Produce Green Hydrogen from Olivine and Seawater? By Ultrasound

**DOI:** 10.1002/cssc.202500627

**Published:** 2025-07-09

**Authors:** Sergey I. Nikitenko, Tony Chave

**Affiliations:** ^1^ Institut de Chimie Separative de Marcoule ICSM, Univ. Montpellier, CEA, CNRS, ENSCM 30207 Marcoule France

**Keywords:** green hydrogen, olivine, seawater, sonochemistry, ultrasound

## Abstract

Green hydrogen is a pillar for achieving global decarbonization and the reduction of greenhouse gas emissions. Here, a new, nature‐inspired process for green hydrogen production using virtually unlimited natural resources is reported. Olivine, the most abundant mineral in the Earth's upper mantle, is key to this process. It is found that 20 kHz ultrasound accelerates hydrogen production from olivine suspensions in seawater under near‐ambient conditions by almost 3000 times compared to the hydrothermal process. Strong mechanical stirring does not lead to hydrogen evolution in the temperature range of 40–90 °C. The striking effect of ultrasound is attributed to acoustic cavitation, which provides depassivation of the olivine surface, fragmentation of olivine particles, and local transient heating caused by collapsing bubbles. In principle, ultrasonic activation of the olivine/seawater system enables on‐demand hydrogen production.

## Graphical abstract

Power ultrasound provides efficient production of green hydrogen from olivine and seawater under near‐ambient conditions. The sonochemical alteration of olivine involves particle fragmentation, the removal of the passivating layer, and local, transient heating in the vicinity of collapsing bubbles. The ultrasonically driven process is suitable for on‐demand hydrogen production.

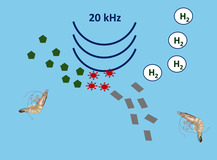

## 1 Introduction

Green hydrogen plays a key role not only for clean energy storage, but also for the decarbonization of various industrial processes such as steelmaking, chemical synthesis, refinery processes, etc.^[^ ^1^ ^]^ Currently, green hydrogen is primarily produced through electrocatalytic water splitting using electricity from renewable sources. Despite its potential as a clean and sustainable method of hydrogen production, water electrolysis is hindered by harsh operating conditions, expensive electrocatalysts, the limited durability of electrolytic cells, and significant freshwater consumption. These factors have slowed its widespread adoption.^[^ ^2^, ^3^ ^]^ Here, we present a new nature‐inspired approach to produce green hydrogen using the reaction of olivine with seawater induced by high‐power ultrasound at near‐ambient conditions.

Olivine is a natural silicate‐type mineral with an approximate composition of (Mg_x_Fe_2−x_)SiO_4_, where x ≈ 1.8–1.9, and featuring different minor substitutions. It is widely believed to be the most abundant mineral in the Earth's upper mantle. Olivine sand serves as a versatile industrial material in metal castings, soil amendment, construction, and water filtration, and its global production is estimated to reach nine million metric tons annually.^[^ ^4^ ^]^ On the other hand, olivine is thermodynamically unstable in aqueous solutions even at ambient temperature. The aqueous alteration of olivine, called serpentinization, leads to the formation of serpentine, Mg_3_Si_2_O_5_(OH)_4_, and brucite, Mg(OH)_2_, with Fe(II)/Fe(III) inclusions.^[^ ^5^, ^6^ ^]^ In the presence of CO_2_, serpentinization of olivine is also accompanied by the formation of magnesite, MgCO_3_, and amorphous silica, SiO_2_.^[^ ^6^, ^7^ ^]^ In addition, thermodynamic calculations have shown that in anoxic conditions water enables oxidation of Fe(II) incorporated into olivine at T ≤ 315 °C producing hydrogen and magnetite:^[^ ^8^ ^]^

(1) $${\left(\text{Mg},\text{Fe}\right)}_{2}{\text{SiO}}_{4}+{\text{nH}}_{2}O\to x{\left(\text{Mg},{\text{Fe}}^{\text{II}},{\text{Fe}}^{\text{III}}\right)}_{3}{\left(\text{Si},{\text{Fe}}^{\text{III}}\right)}_{2}O_{5}{\left(\text{OH}\right)}_{4}+y\left(\text{Mg},{\text{Fe}}^{\text{II}}\right){\left(\text{OH}\right)}_{2}+{\text{zFe}}_{3}O_{4}+\left(n-2x-y\right)H_{2}$$

The stoichiometric coefficients *n*, *x*, *y,* and *z* in this general equation strongly depend on iron partitioning among the reaction products.^[^ ^9^ ^]^ The reaction (1) is thought to produce ≈80% of the naturally occurring (white) hydrogen.^[^ ^10^ ^]^ However, the reaction (1) proceeds too slowly for efficient green hydrogen production. McCollom et al. reported the rate of H_2_ generation during hydrothermal olivine alteration at temperatures between 200 and 230 °C and a pressure of ≈350 bar.^[^ ^11^ ^]^ They found that the rate increased from 2 10^−3^ to 35 10^−3^ μmol g^−1^ h^−1^ as the pH of the solution increased from 7.8 to 12.5. In the presence of CO_2_, Wang et al. found an H_2_ formation rate of about 1–3 μmol g^−1^ h^−1^ at *T* = 225–300 °C and *P* = 100 bar.^[^ ^12^ ^]^ Kinetic constraints of the reaction (1) are attributed to several factors. First, olivine is a nonporous material and only about 0.1% of Fe(II) at the particle surface is available for H_2_ generation.^[^ ^13^ ^]^ It is therefore suggested that a dense network of surface cracks plays an important role in the evolution of H_2_ from olivine. At a second glance, thin layers of amorphous silica^[^ ^7^ ^]^ and Mg(OH)_2_ with incorporated Fe(II) and Fe(III) moieties^[^ ^8^ ^]^ formed during olivine weathering severely limit H_2_ evolution.

We hypothesized that power ultrasound could be used to overcome both constraints on olivine serpentinization kinetics. Ultrasonic treatment is known as a powerful tool for activating solid surfaces.^[^ ^14^ ^]^ The physicochemical effects of ultrasound, called sonochemistry, refer to acoustic cavitation. The propagation of ultrasonic waves in liquids leads to the formation of gas‐ and vapor‐filled microbubbles, which implode violently after several oscillation cycles. The implosive collapse of the cavitation bubbles produces harsh transient conditions within the bubbles and various mechanical effects in solutions or at solid/liquid interfaces. Collapsing bubbles generate microjets with a velocity of a few hundred meters per second and shock waves with a local peak pressure of several GPa in close vicinity of the bubble.^[^ ^15^ ^]^ In addition, the energy released after bubble collapse causes transient (<2 μs) overheating of the liquid shell about 200 nm around the bubbles to several hundred Kelvins.^[^ ^16^ ^]^ It is worth noting that the cavitation erosion is much stronger at low‐frequency ultrasound (16–60 kHz) compared with high ultrasonic frequency (200 kHz–1 MHz).^[^ ^17^ ^]^ Ultrasonic treatment with 20 kHz ultrasound provides efficient erosion and fracture of bulk metals and alloys,^[^ ^18^ ^]^ glasses,^[^ ^19^ ^]^ and refractory ceramics.^[^ ^20^, ^21^ ^]^ It is interesting to note that the combination of ultrasound and complexing agents, like chloride or nitrate ions, improves the removal of passivating layers from the material surface leading to a significant acceleration of metal leaching from a solid matrix and dissolution of solids.^[^ ^22^, ^23^ ^]^ Acoustic cavitation induces also a splitting of water molecules leading to the release of H_2_.^[^ ^14^ ^]^ However, the efficiency of H_2_ sonochemical production is quite low. Recently, Mondal et al. reported significant improvement in H_2_ production from distilled water in the presence of piezoelectric MoS_2_ particles submitted to power ultrasound.^[^ ^24^ ^]^ It should be noted that the performance of the process is decreased in seawater. In this work, we found that 20 kHz ultrasound accelerates H_2_ emission from suspensions of olivine in seawater by about 3000 times at near‐ambient conditions compared to hydrothermal treatment without ultrasound.

## 2 Results and Discussion

In the experiments, we used olivine sand provided by American Elements with an average composition (Mg_0.88_Fe_0.12_)_2_SiO_4_ close to San Carlos olivine.^[^ ^25^ ^]^ The detailed chemical composition of the sand sample obtained with scanning electron microscopy/energy dispersive X‐ray (SEM/EDX) analysis is listed in Table 1S, Supporting Information. The sand was milled and sieved to recover the <150 μm‐sized fractions. The specific surface area of the powder was determined to be 1.8 m^2^ g^−1^ using a ten‐point Brunauer–Emmett–Teller (BET) method with Kr as an adsorbent gas. Seawater was collected from the Mediterranean sea (France) and filtered through 0.2 μm filter. For comparison, some experiments were carried out in Milli‐Q water (18.2 MΩ cm) and 0.49 M NaCl aqueous solutions to approximate seawater salinity. In a typical run, 75 mL of liquid mixed with 2 g of olivine powder was treated using a 20 kHz ultrasonic probe. Pure argon was bubbled at a rate of 60 mL min^−1^ for about 30 min before and during the ultrasonic treatment. The thermosttated sonochemical reactor is shown in Figure S1, Supporting Information. The temperature in the reactor during the process was maintained at a steady state of 70–75 °C. The specific acoustic power, *P*_ac_, transmitted to the solution was measured by the conventional thermal probe method. The formation of gaseous products was monitored online in the outlet gas by mass spectrometry. Further experimental details are described in Supporting Information.

**Figure 1:**
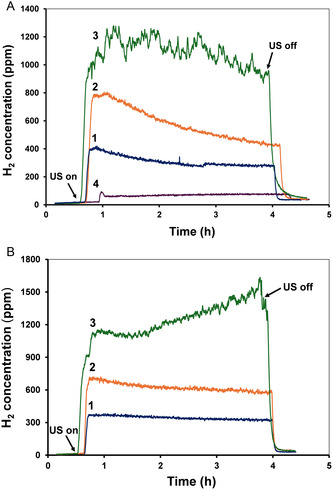
Typical emission profiles of H_2_. A) Seawater. 1. pH = 9.0, *P*_ac_ = 60 W, 2. pH = 9.1, *P*_ac_ = 109 W, 3. pH = 12.1, *P*_ac_ = 109 W, 4. Seawater without olivine, pH = 8.1, *P*_ac_ = 109 W, emission of CO_2_ and CH_4_ is not observed. B) Milli‐Q water and 0.49 M NaCl aqueous solutions. 1. Milli‐Q, pH = 9.8, *P*_ac_ = 109 W, 2. 2. 0.49 M NaCl, pH = 9.1, *P*_ac_ = 109 W, 3. 0.49 M NaCl, pH = 12.3, and *P*_ac_ = 12.3.

In the preliminary experiments, it was found that vigorous mechanical stirring of powdered olivine in Milli‐Q or seawater does not produce H_2_ in the temperature range of 40–90 °C. On the other hand, exposure to ultrasound induces strong H_2_ emission for both seawater and aqueous suspensions (**Figure** **1**). The mass spectrometric analysis also revealed the emission of small amounts of CO_2_ and CH_4_ (Figure S2 and Figure S3, Supporting Information respectively). At pH about 9 (Figure 1A,B curves 1 and 2), the H_2_ concentration increases rapidly in the first stage of the process, reaching a maximum value, followed by a progressive decrease in H_2_ emission, which could be attributed to the secondary passivation of the olivine surface. On the other hand, at pH about 12 (Figure 1A,B curve 3), the evolution of H_2_ strongly increases and repassivation is no longer observed in seawater and in salted Milli‐Q water. Instead, the H_2_ emission shows a spike‐shaped profile, which could possibly be explained by the ultrasonically induced failure of olivine particles leading to the formation of fresh solid surfaces more active in the H_2_ evolution process. It is worth noting that H_2_ is also produced in the investigated systems by the sonochemical splitting of water molecules, but its efficiency is negligible compared to H_2_ production from olivine and water (Figure 1A, curve 4).

The values of the average integrated rate (AIR) of gaseous product formation, as calculated from the kinetic curves shown in Figure 1 and summarized in **Table** **1**, highlight the main factors influencing H_2_ production kinetics. We found that increasing the acoustic power from 60 to 109 W (runs 1–3) caused a significant increase in the H_2_ production rate. This confirms the primordial role of cavitation in the overall process. It is interesting that the AIR values for H_2_ in seawater (run 3) and in salted H_2_O (run 6) are larger than in pure H_2_O (run 5). This observation is consistent with the increased olivine weathering in seawater reported by several authors.^[^ ^26^, ^27^ ^]^ In the systems studied, the beneficial effect of water salinity can be attributed to the surface complexation of Mg(II) ions with chloride anions, which would facilitate the alteration of olivine by water. Raising the pH to around 12 (runs 4 and 7) dramatically accelerates the production of H_2_. A similar phenomenon was observed during hydrothermal serpentinization of olivine.^[^ ^11^ ^]^ It was suggested that the greater rate of H_2_ production at higher pH is attributed to the increased overall rate of olivine alteration. The inductively coupled plasma optical emission spectrometry (ICP‐OES) analysis of solutions obtained during ultrasonic treatment, summarized in **Table** **2**, clearly shows the increase in dissolved silica concentration at higher pH. This observation suggests that the increased rate of H_2_ production is due to the combined effects of power ultrasound and alkaline conditions on removing the SiO_2_ passivation layer formed during the initial stage of the process. Such an effect leads to an improvement of the H_2_ emission kinetics of about 3000 times compared to the hydrothermal process under silent conditions. It is interesting to note that the addition of citric acid resulted in an acceleration of olivine dissolution (Table 2) but without a significant effect on the H_2_ production rate (run 8, Table 1). The H_2_ emission profile in the presence of 0.02 M citric acid is shown in Figure S4, Supporting Information. This result can be explained by the strong complexation of Fe(II) with citrate anions (log*β*_1_ = 8.62), which retards Fe(II) oxidation by water.^[^ ^28^ ^]^

**Table 1:** AIR of gaseous products formation in studied systems. SW–seawater, OL–olivine powder. *f* = 20 kHz, *T* = 70–75 °C, Ar–60 mL min^−1^.

Run	**System**	**pH**	***P***_**ac**_ **[W]**		AIR [μmol g^−1^ h^−1^]	
				H_2_	CO_2_	CH_4_
**1**	SW‐OL	9.0 ± 0.1	60	16 ± 2	2.1 ± 0.5	≈0
**2**	SW‐OL	9.0 ± 0.1	71	25 ± 2	2.6 ± 0.5	≈0
**3**	SW‐OL	9.1 ± 0.1	109	53 ± 4	3.0 ± 0.5	traces
**4**	SW‐OL	12.1 ± 0.2a)	109	96 ± 5	≈0	0.18 ± 0.09
**5**	H_2_O‐OL	9.8 ± 0.2	109	28 ± 2	0.8 ± 0.2	≈0
**6**	0.49M NaCl‐OL	9.1 ± 0.2	109	50 ± 6	4.5 ± 0.6	traces
**7**	0.49M NaCl‐OL	12.3 ± 0.2a)	109	105 ± 7	≈0	0.31 ± 0.08
**8**	SW‐0.02M HCit‐OLb)	7.3 ± 0.2	109	48 ± 5	traces	8.1 ± 1.1
	0.49 MNaCl‐OLc) 230 °C, 350 bar, silent	12.5	–	3.3 10^−2^	–	–

a) pH was adjusted by NaOH addition

b) HCit–citric acid.

c) Data of McCollom et al.^[^ ^11^ ^]^

**Table 2:** Solution compositions obtained under silent conditions and during ultrasonic treatment at *P*_ac_  = 109 W for 4 h. *f* = 20 kHz, *T* = 70–75 °C, Ar–60 mL min^−1^. SW–seawater, OL–olivine powder.

System	pH		Concentration [ppm] ± 10%	
		**Si** a)	Mg	Fe
H_2_O‐OL silent	10.2 ± 0.2	6.0	13.8	≈0
H_2_O‐OL ultrasound	9.8 ± 0.1	3.3	10.7	≈0
0.49M NaCl‐OL silent	12.1 ± 0.1	41.8	≈0	≈0
0.49M NaCl‐OL ultrasound	12.3 ± 0.2	58.1	≈0	≈0
SW‐OL silent	9.2 ± 0.1	12.3	1314b)	≈0
SW‐0.02M HCitc)‐OL ultrasound	7.3 ± 0.2	60.8	1751	88.6

a) Si corresponds to dissolved species of silica (SiO_2_ and HSiO_3_^−^),

b) Mg originated from seawater,

c) HCit–citric acid.

In general, the emission of CO_2_ observed during ultrasonic treatment at pH about 9 (Table 1) is not reported in the studies of natural weathering of olivine. It's rather the opposite, olivine is considered as a potential promising candidate for CO_2_ sequestration.^[^ ^7^, ^26^, ^27^, ^29^ ^]^ In the case of the ultrasonically driven process, the formation of CO_2_ can be attributed to the decomposition of nesquehonite (MgCO_3_·3H_2_O), which is usually present on the surface of hydrated olivine‐like materials.^[^ ^30^ ^]^ It is known that under hydrothermal conditions (*T* > 100 °C and *P* ≈ 100 bar) MgCO_3_·3H_2_O undergoes partial decarbonation with the formation of hydromagnesite (4MgCO_3_·Mg(OH)_2_·4H_2_O).^[^ ^31^ ^]^ In the process studied here, such high transient local temperatures and pressures can be generated by the cavitation bubbles imploded in close vicinity to the olivine surface.^[^ ^32^ ^]^ This conclusion correlates with the observation of CH_4_ formation during ultrasonic alteration of olivine. It has been previously reported that the release of CH_4_ during the hydrothermal serpentinization of olivine in the presence of dissolved CO_2_ is due to the methanation of CO_2_ with H_2_ (Sabatier reaction) catalyzed by iron oxides also formed as a product of olivine weathering.^[^ ^33^ ^]^ This reaction is thermodynamically favorable at *T* ≈ 300 °C^[^ ^34^ ^]^ indicating that the mechanism of sonochemical alteration of olivine involves not only mechanical effects but also the strong, transient local heating produced by acoustic cavitation. It should be noted, however, that the rate of CO_2_ and CH_4_ formation is much lower than that of H_2_. The relatively higher rate of CH_4_ formation in the presence of citric acid is likely due to its sonochemical degradation rather than a reaction with olivine.

**Figure 2:**
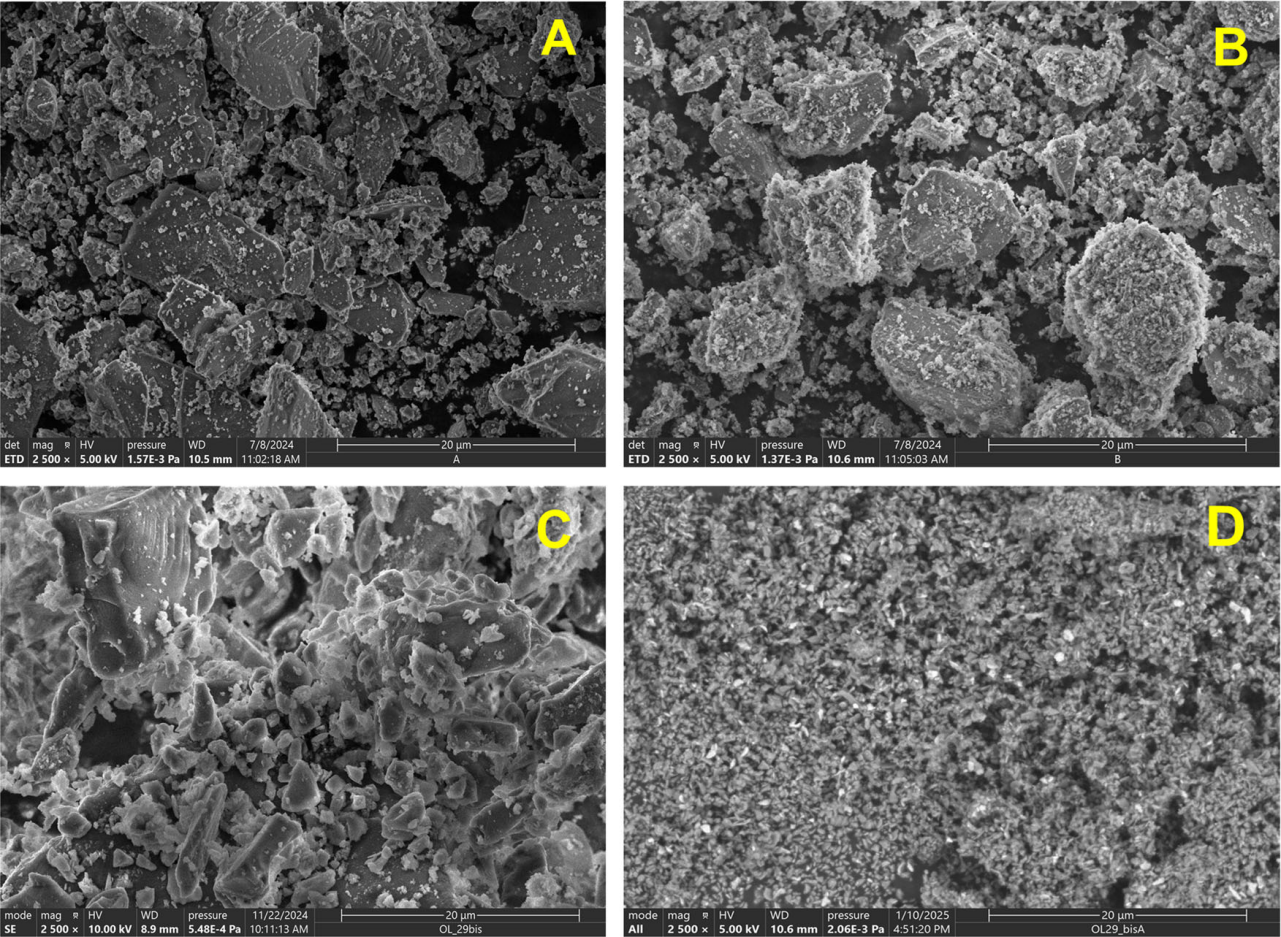
SEM images. A) Pristine olivine. B) Olivine treated in seawater at pH = 9.1 for 8 h. C,D) Olivine treated in 0.49 NaCl aqueous solution at pH = 12.1, **C**.–4 h, **D**.–8 h. *P*_ac_ = 109 W, Scale bar is 20 μm.

Analysis of solid products provided further insight into the mechanism of ultrasonic olivine alteration. It is worth noting that the color of olivine changes rapidly from milky white to deep gray during the ultrasonic treatment as shown in Figure S5, Supporting Information. The SEM images depicted in **Figure** **2** illustrate the strong effect of acoustic cavitation on olivine particle size and morphology. At pH = 9.1, the particles exhibit surface pitting corrosion rather than significant particle fragmentation (Figure 2A,B). However, at pH = 12.1, SEM image reveals a conchoidal fracture of brittle olivine particles increasing with the time of ultrasonic treatment (Figure 2C,D). Such a change in particle morphology correlates with the increase in H_2_ production rate in more alkaline solutions.

**Figure 3:**
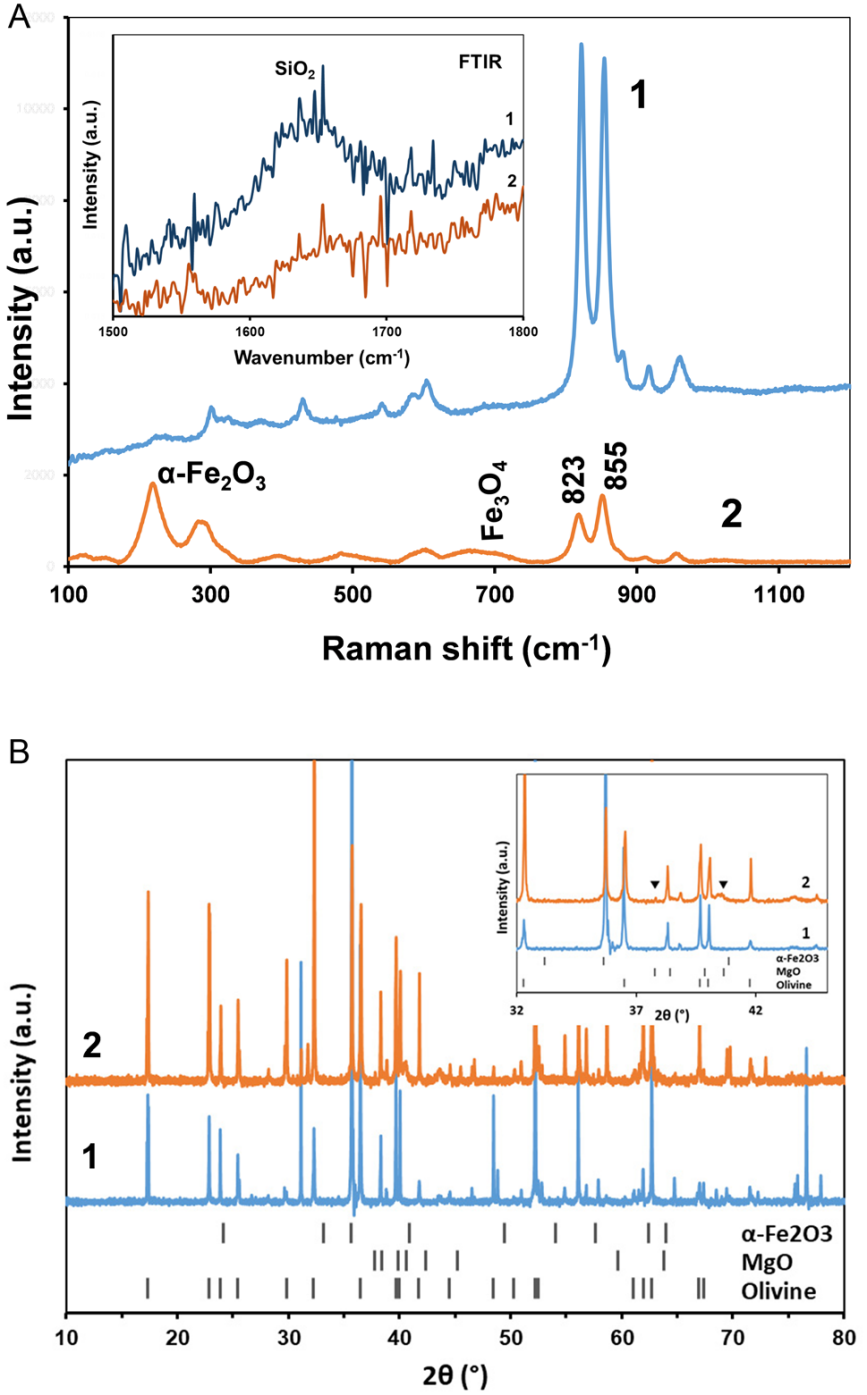
Raman, FTIR spectra, and XRD patterns. A) Raman and FTIR spectra (inset). B) XRD patterns. 1. Pristine olivine, 2. Olivine treated in 0.49 M NaCl aqueous solution at pH = 12.1. *P*_ac_ = 109 W, time of ultrasonic treatment is 8 h. Solids were rinsed with water and dried prior to analysis. (▾) MgO patterns.

Raman spectrum of pristine olivine (**Figure** **3**A) shows a dominant doublet at 823 and 855 cm^−1^ typical for olivine minerals and attributed to the internal stretching vibrational modes of the SiO_4_^4−^ tetrahedral.^[^ ^34^ ^]^ The spectrum of ultrasonically treated olivine exhibits a similar doublet, but with a larger relative intensity of 855 cm^−1^ band compared to the untreated sample, clearly indicating the depletion of the olivine structure with Fe(II).^[^ ^34^ ^]^ Surprisingly, this Raman spectrum reveals only a very weak band of Fe_3_O_4_ (660–690 cm^−1^), as well as the absence of the Mg(OH)_2_ band at 3650 cm^−1^ and lizardite (serpentine) band at 3600–3700 cm^−1^ (Figure S6, Supporting Information), identified as the products of olivine hydrothermal serpentinization according to the equation (1). Instead, the Raman spectrum of olivine altered by ultrasound in alkaline solutions shows the characteristic bands of hematite, *α*‐Fe_2_O_3_, at 222 and 281 cm^−1^. The weak IR band centered at 1650 cm^−1^ in the FTIR spectrum of pristine powdered olivine (inset in Figure 3A) can be assigned to the hydrated amorphous SiO_2_ layer at the olivine surface.^[^ ^35^ ^]^ This band disappeared after ultrasonic treatment in alkaline solutions, which is consistent with the kinetic data of olivine depassivation under these conditions. Figure 3B shows the X‐ray diffraction (XRD) diagram of the ultrasonically treated olivine, which is dominated by olivine patterns (JCPDS 77‐1028). This indicates that removing Fe(II) does not alter the orthorhombic symmetry of the olivine crystalline structure. Additionally, the XRD analysis reveals the presence of MgO (JCPDS 80‐4186) and possibly *α*‐Fe_2_O_3_. However, its patterns strongly overlap with those of olivine and MgO (41‐43 2*θ*°). The characteristic pattern of serpentine (lizardite) at 12 2*θ*° (JCPDS 14‐177) is not observed in the XRD diagram of sonicated olivine, which is consistent with the Raman spectra.

The above results suggest a significant difference in the mechanisms of ultrasonic and silent hydrothermal alteration of olivine. In fact, the sonochemical process does not lead to serpentinization, but rather to the depassivation and fracturing of olivine particles, followed by the oxidative leaching of Fe(II) and the emission of H_2_. We suggested that this difference is due to two factors: the strong mechanical effects of shock waves and microjets produced by bubble collapse, and the strong yet transient (lasting several μsec), local heating of the olivine particle surface at the moment of bubble implosion. In anoxic conditions, noticeable hydrothermal conversion of olivine to serpentine followed by Fe_3_O_4_ formation requires several days of treatment in a closed system.^[^ ^6^ ^]^ In the case of ultrasonic activation, Fe_3_O_4_ is likely to be formed as an intermediate product with its further rapid oxidation to *α*‐Fe_2_O_3_. Several experimental results have demonstrated that, at temperatures above 200 °C, hydrothermal leaching of Fe(II) from Fe_3_O_4_ leads to the formation of *α*‐Fe_2_O_3_ without H_2_ emission.^[^ ^36^, ^37^ ^]^ Local heating caused by the bubble collapse can induce this process. It should also be noted that, unlike the hydrothermal process, the sonochemical alteration of olivine studied in this work is an open system. Therefore, the reduction of *α*‐Fe_2_O_3_ back to Fe_3_O_4_ by released H_2_ seems unlikely.

## 3 Conclusion

This study reports on an innovative process for producing green hydrogen from virtually unlimited natural resources, such as seawater and olivine, using power ultrasound. This process does not require scarce noble metals or specific harsh conditions. Ultrasonic activation accelerates hydrogen emission from olivine/seawater suspensions by about 3000 times compared to traditional hydrothermal treatment. This striking result is achieved due to the unique set of conditions provided by acoustic cavitation, such as efficient mechanochemical depassivation and fragmentation of olivine particles, as well as strong, transient local heating caused by the collapse of cavitation bubbles. Our results also show that the products of ultrasonically‐driven olivine interaction with water are different from the hydrothermal process. Instead of brucite, Mg(OH)_2_, and magnetite, Fe_3_O_4_, ultrasonic treatment leads to magnesium oxide, MgO, and hematite, *α*‐Fe_2_O_3_. In addition, the formation of serpentine (Mg_3_Si_2_O_5_(OH)_4_), is not observed under power ultrasound. It should be emphasized that, unlike water electrolysis, the sonochemical process is more efficient in seawater than in pure water. In the absence of power ultrasound, any hydrogen is formed under the studied conditions, offering the possibility of producing green hydrogen on demand instead of using costly technologies to store large quantities of hydrogen.

## Conflict of Interest

The authors declare no conflict of interest.

## Supporting information

Supplementary Material

## Data Availability

Data sharing is not applicable to this article as no new data were created or analyzed in this study.
