# Supplementary material for: How to Produce Green Hydrogen from Olivine and Seawater? By Ultrasound
Source: ChemSusChem. 2025 Jul 9;18(18):e202500627. doi: 10.1002/cssc.202500627 (PMC12456393; doi:10.1002/cssc.202500627)
Supplement: Supplementary file 1 — Supplementary Material [file CSSC-18-e202500627-s001.pdf]

Supporting Information

How to produce green hydrogen from olivine and seawater? By ultrasound

Sergey I. Nikitenko, Tony Chave

Table S1. Chemical composition of the olivine powder obtained by SEM/EDX analysis.

Element	Line series	Atom. %	1σ (%)
Carbon	6 K	3.7	6
Oxygen	8 K	54.1	4
Magnesium	12 K	23.7	4
Silicon	14 K	14.1	3
Iron	26 K	8.3	2.3
Aluminum	13 K	traces	
Sulfur	16 K	0.01	57
Nickel	28 K	0.12	9
Copper	29 K	0.12	11
Calcium	20 K	0.06	11
Manganese	25 K	0.04	16
**Total**		104	

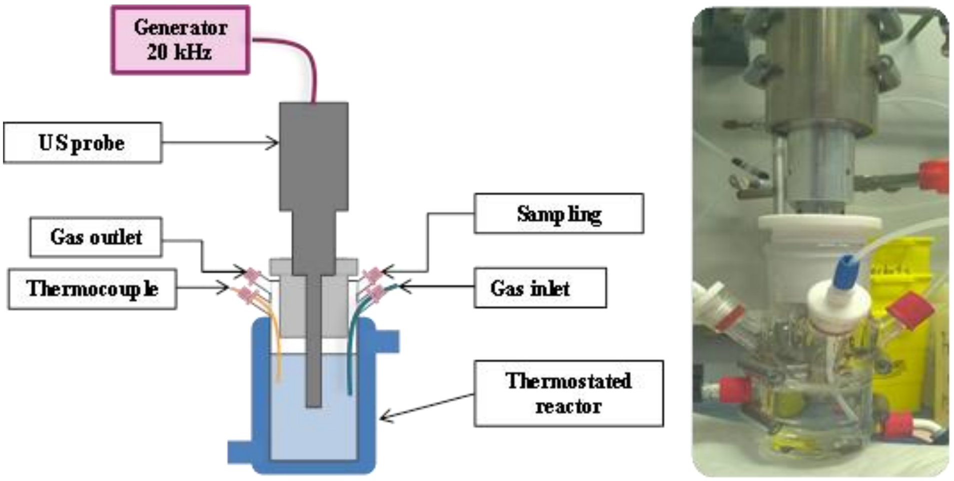

Figure S1. Graphical sketch and image of the sonochemical reactor.

Sonochemical experiments

Sonochemical experiments were performed in a thermostated reactor shown in Figure S1. In a typical run, 75 mL of liquid mixed with 2 g of olivine was treated with 1 cm^2 ultrasonic probe made of Ti-6Al-4V alloy. The piezoelectric transducer was supplied by a 20 kHz generator (Vibra-Cell VCX 750 W). The probe was immersed reproducibly 3 cm from the bottom of the reaction vessel. The temperature in the reactor during the process was maintained at a steady-state temperature of 70-75 °C. Pure Ar (Air Liquide, O_2 <1 ppm) was bubbled at a rate of 60

mL min^{-1} for about 30 min before and during the ultrasonic treatment. The gas flow rate was controlled by a volumetric flowmeter. The specific acoustic power, P_{ac}, transmitted to the solution was measured by conventional thermal probe method.

After the ultrasonic treatment solids have been removed by centrifugation, rinsed with Milli-Q water and dried at 70 °C under reduced pressure.

Analysis

Gaseous products were monitored online in the outlet gas with Prima BT Benchtop mass spectrometer (Thermo Scientific) by Multiple Ion Monitoring (MIM) system. The concentrations of H_2, CO_2, and CH_4 were quantified using external calibration curves prepared with standard gas mixtures in argon (Air Liquide). Then the formation rate of the products was calculated using a known argon flowrate.

Powder X-Ray Diffraction (XRD) diagrams were recorded with the use of a Bruker D8 Advance X-ray diffractometer equipped with a linear Lynx-eye detector (Cu $K\alpha_{1,2}$ radiation, λ = 1.54184 Å). XRD patterns were collected between 10° and 90° (θ-2θ mode) at room temperature, with a step size of $\Delta(2\theta) = 0.01°$ and a counting time of 1s step^{-1}. XRD data were corrected with Cu-$K\alpha_2$ radiation stripping and background subtraction.

Raman spectra were obtained using a Horiba – Jobin Yvon Aramis apparatus equipped with an edge filter and a Nd:YAG laser working at 532 nm with a power of 39 mW. The laser beam was focused with an Olympus BX41 microscope on the sample deposited on a glass lamella. Before the analysis of the sample, the apparatus was calibrated with a silicon wafer, using the first-order Si line at 520.7 cm^{-1}. Each spectrum was averaged from 5 parallel runs.

FT-IR spectra were recorded using Spectrum 100 (Perkin Elmer) spectrometer equipped with UATR unit. Each spectrum was averaged from 5 parallel runs.

Scanning electron microscopy measurements coupled with energy dispersive X-ray analysis (SEM/EDX) were done with a QUANTA FEG 200 ESEM scanning microscope without surface metallization.

Inductively coupled plasma optical emission spectroscopy (ICP-OES) was used for quantitative analysis of Mg, Si, and Fe in solutions with a Spectro Arcos apparatus (Spectro Analytical Instruments GmbH) equipped for axial plasma observation. After filtration, the samples were diluted using 0.3 M HNO_3. The concentrations of the elements were evaluated using calibration curves obtained with certified standard solutions of corresponding elements (SCP Science).

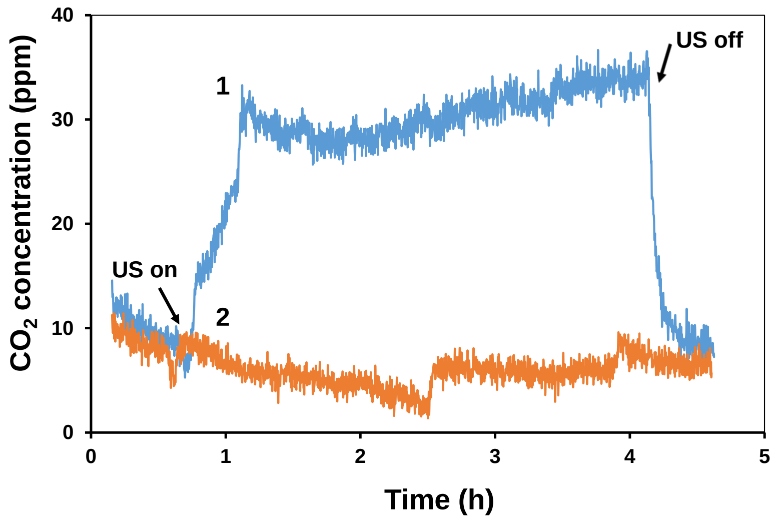

Fig S2. CO_2 emission profiles during sonication of olivine in seawater at pH = 9.1 (1) and pH = 12.1 (2).

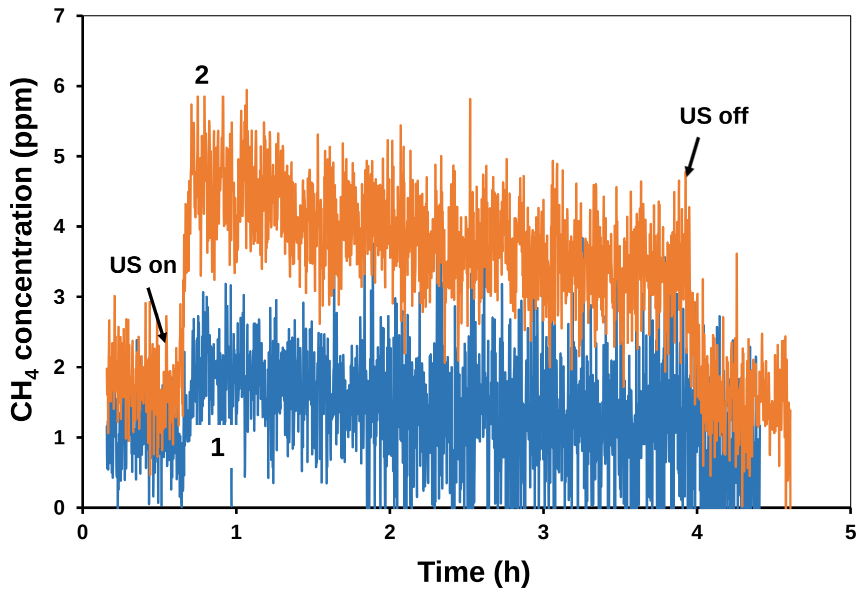

Fig S3. CH_4 emission profiles during sonication of olivine in seawater at pH = 9.1 (1) and pH = 12.1 (2).

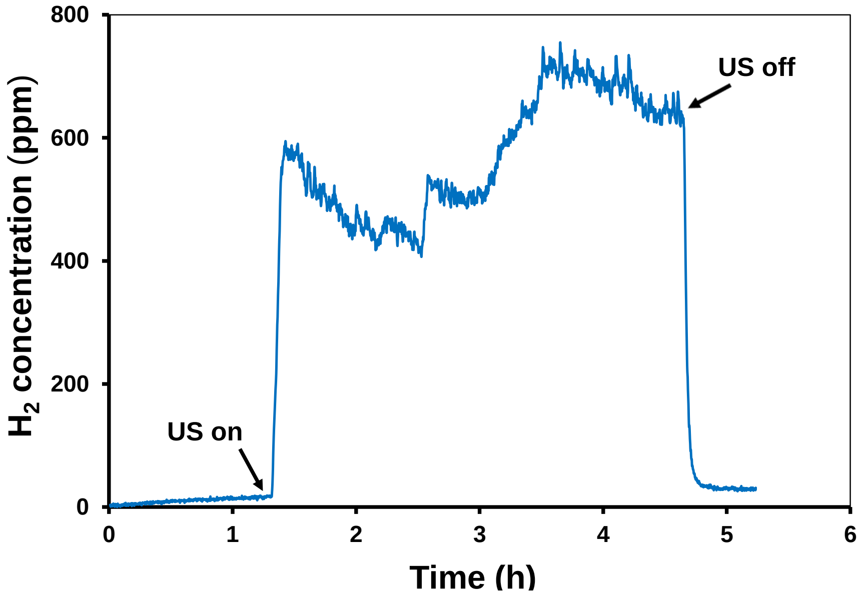

Fig S4. H_2 emission profile during sonication of olivine in seawater in the presence of 0.02M citric acid, pH = 7.3.

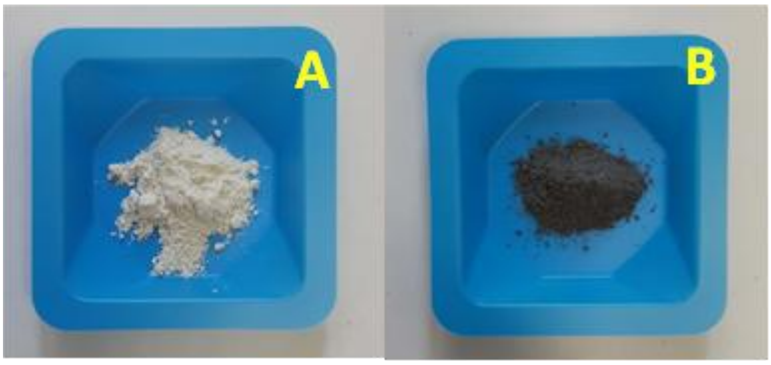

Fig S5. Images of olivine powder before (A) and after (B) ultrasonic treatment in seawater. Powders prepared in pure water and 0.49 M NaCl solutions are similar in color.

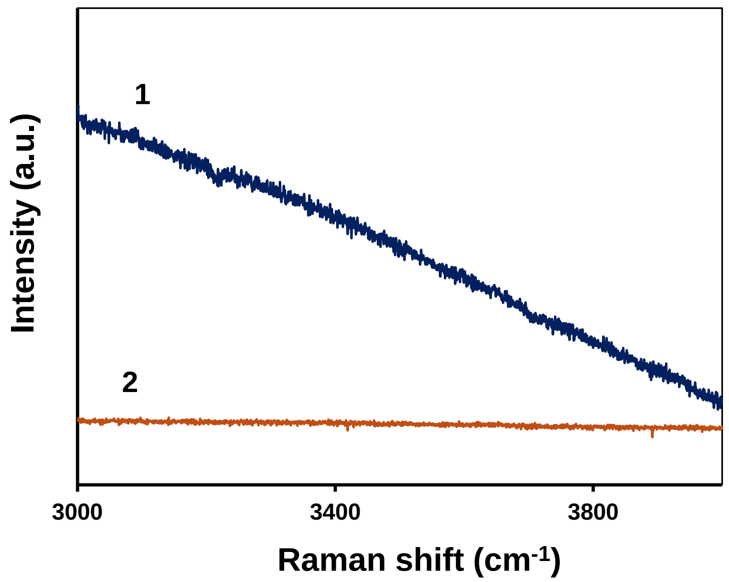

Fig. S6. Raman spectra in the range of 3000 – 4000 cm^{-1}. 1. Pristine olivine, 2. Olivine treated in 0.49 M NaCl aqueous solution at pH = 12.1. P_{ac} = 109 W, time of ultrasonic treatment is 8 h.
